# The Use of Automated Bioacoustic Recorders to Replace Human Wildlife Surveys: An Example Using Nightjars

**DOI:** 10.1371/journal.pone.0102770

**Published:** 2014-07-16

**Authors:** Mieke C. Zwart, Andrew Baker, Philip J. K. McGowan, Mark J. Whittingham

**Affiliations:** 1 Newcastle University, School of Biology, Newcastle upon Tyne, United Kingdom; 2 Baker Consultants Ltd, Matlock, United Kingdom; University of Durham, United Kingdom

## Abstract

To be able to monitor and protect endangered species, we need accurate information on their numbers and where they live. Survey methods using automated bioacoustic recorders offer significant promise, especially for species whose behaviour or ecology reduces their detectability during traditional surveys, such as the European nightjar. In this study we examined the utility of automated bioacoustic recorders and the associated classification software as a way to survey for wildlife, using the nightjar as an example. We compared traditional human surveys with results obtained from bioacoustic recorders. When we compared these two methods using the recordings made at the same time as the human surveys, we found that recorders were better at detecting nightjars. However, in practice fieldworkers are likely to deploy recorders for extended periods to make best use of them. Our comparison of this practical approach with human surveys revealed that recorders were significantly better at detecting nightjars than human surveyors: recorders detected nightjars during 19 of 22 survey periods, while surveyors detected nightjars on only six of these occasions. In addition, there was no correlation between the amount of vocalisation captured by the acoustic recorders and the abundance of nightjars as recorded by human surveyors. The data obtained from the recorders revealed that nightjars were most active just before dawn and just after dusk, and least active during the middle of the night. As a result, we found that recording at both dusk and dawn or only at dawn would give reasonably high levels of detection while significantly reducing recording time, preserving battery life. Our analyses suggest that automated bioacoustic recorders could increase the detection of other species, particularly those that are known to be difficult to detect using traditional survey methods. The accuracy of detection is especially important when the data are used to inform conservation.

## Introduction

Information on where species occur and in what numbers are important for an increasing variety of reasons, whether it is for understanding environmental change, assessing and monitoring conservation status or as part of a legislative or policy process. A variety of approaches, sampling designs and field protocols have been developed to meet such needs, such as point counts, transect counts and mapping [1]–[4]. The increasing demand for these data, in particular for informing environmental impact assessments (EIAs), means that new opportunities should be explored to align technical developments with sound sampling design and appropriate field protocols. This is critical if cost-effective and accurate ways of providing data on species occurrence and abundance are to be found. This need is most evident for species that are considered threatened and which are often described as cryptic because their behaviour or ecology substantially reduces their detectability during standard surveys [5]. The European nightjar (*Caprimulgus europaeus*), which is listed on the Annex I Birds Directive [6] and is a Biodiversity Action Plan Priority Species in the UK, presents precisely these challenges. In particular, nightjars are difficult to detect as they are nocturnal and very well camouflaged. The traditional way to survey for them is via surveys at either dusk or dawn. Their vocalisation is less complex than many other bird songs, consisting primarily of a series of pulses, and thus lends itself to automated classification [7]. We therefore explored whether automated acoustic recorders offer a cost-effective way of increasing the reliability of surveys when compared with current approaches.

Audio recording has already been used as a replacement for point counts [8]–[11] or for identifying individuals [12], [13]. However, in the case of point counts, the potential for recorders to be deployed for longer periods has not been investigated. In addition, as computer technology has advanced and faster computers have become more readily available there is potential for using automatic call recognition for the audio recordings collected, where the species that are on the audio recordings are automatically classified based on an algorithm. Automated classification has been successfully applied to detect a range of species including woodpecker spp. [9] and antbird spp. [14].

In this study we examined the utility of automated bioacoustic recorders and the associated classifying software as a way to survey for wildlife, using the nightjar as an example. We compared detection by traditional surveys, described by Gilbert et al. [15], with results obtained using bioacoustic recorders. We end by making recommendations for the best use of these recording devices for nightjars and other species.

## Materials and Methods

### Ethics statement

Permission to carry out the fieldwork at these sites was obtained from the Forestry Commission under permit number 144/12. As this was a purely observational study, no specific ethical approval was needed.

### Study sites

We chose two Forestry Commission sites in Northumberland, UK, where nightjars were previously recorded: Slaley Forest (geographic coordinates of the central point of the site: 54°53′11″N, 2°5′21″W) and Fourlaws (geographic coordinates of the central point of the site: 55°8′50″N, 2°7′11″W). These sites comprised a mixture of different age stands of coniferous woodland, heather moorland, and a small amount of deciduous woodland around the edges of the conifer stands.

### Traditional site surveys

Between mid-June and the end of July 2012, which is during the breeding season of the nightjar [15], we performed four surveys at each site. Each survey was separated by a two-week interval. Surveys began at dusk on clear nights with low to no wind. Survey methods followed the methods described in Gilbert et al. [15]. In short, human surveyors walked a route of 6 km at a speed of 3–4 km/h and stopped every few minutes to listen for churring or calling nightjars. The starting point and route of surveys were randomised so that we did not favour a particular area at the same time. When a nightjar was located, its locality and behaviour was recorded with a handheld GPS device (Trimble Juno 3B) which ran ArcPad 10.0.

### Recorders

At each site three full spectrum recorders (SM2+, Wildlife Acoustics Inc.) were placed, one per km^2^ (six in total), during the same period as the traditional site surveys (see Figure S1). The recorders had a microphone on both the left and right side and were attached to a tree. The recorders were set to record throughout the night between 22.00 and 04.30 the following morning on both channels with a gain of +48 dB and sampling rate of 44100 Hz (see Table S1 & S2 for details). Recordings were saved to disk at 30-minute intervals to prevent the loss of an entire evening’s recordings in the event the batteries ran out. These 30 min. segments were saved in a compressed (native.wac) format. Each recorder continued to record until the batteries ran out of power (SM2+ with GPS average 14.1 hours (range 13.5–15), SM2+ without GPS average 25.4 hours (range 23.5–26.5)). This resulted in an average recording period of four nights (range three to five nights). We used Energizer D 1.2V NiMH 2500 mAh rechargeable batteries. These batteries were replaced every week for nine weeks between mid-June and mid-August 2012. The traditional surveys took place at the beginning of week 1, 3, 5 and 7. Recorder 6 was put out at week 4 and recorders 4–6 were taken down after week 7 due to site permission restrictions. All other recorders were active for the nine week duration.

### Recognizer

We used an automated recognizer rather than listening to 1948 hours of recordings (196, 198, 194.5, 142, 145.5 and 98 hours of recordings per channel on recorder 1–6 respectively). Recognizers for churring and for flight calls were generated in Song Scope 4.1.3A (Wildlife Acoustics Inc.). A fast Fourier Transformation (FFT) size of 64 and 50% overlap was used with a frequency range of 500–3000 Hz. These settings were chosen to get a good temporal resolution of the individual pulses that make up the churring of the nightjar. In total 181 suitable sections of recorded nightjar churring were selected from 12 files from two different recorders and loaded in the program as training data for the churring recognizer. The 12 files corresponded to 3.8% of the total number of files where some nightjar churring was detected. On average the sections were of 3.67±2.16 (1SD) seconds in length. For the flight call recognizer 32 flight calls were selected from 15 files from four different recorders. These files were 5.5% of the total number of files where some nightjar flight calling was detected. The sections that were selected were on average of 0.40±0.11 (1SD) seconds in length. When building the recognizer we consulted with Wildlife Acoustics Inc. in order to choose the settings that achieved the best results. Several different configurations of settings were tested on a small sample set of the data (see Table S3 for details on the different configurations tested). This sample set included both positive and negative controls: that is, audio files where it was known that nightjars were churring and flight calling, and also audio files where it was known that no nightjars were churring or flight calling. We chose the final configuration settings in order to achieve the lowest false positive and false negative rates. This criterion resulted in the following settings for building the recognizer for churring: maximum complexity: 16, maximum resolution: 15, sample rate: 8 kHz, FFT size: 64, FFT overlap: ½, frequency minimum: 8, frequency maximum: 18, background filter: 1s, maximum syllable length: 148 ms, maximum syllable gap: 148 ms, maximum song length: 5980 ms, dynamic range: 10 and algorithm: 2.0. The following settings were used for the flight call recognizer: maximum complexity: 16, maximum resolution: 10, sample rate: 8 kHz, FFT size: 256, FFT overlap: ½, frequency minimum: 28, frequency maximum: 88, background filter: 1 s, maximum syllable length: 256 ms, maximum syllable gap: 0 ms, maximum song length: 352 ms, dynamic range: 20, algorithm: 2.0.

### Analysis

#### Extracting data from the recorders

For each compressed wac file the two channels were saved as separate files and converted into a wav file with wac2wav 3.3.0 (Wildlife Acoustics Inc.). A batch process was set up where the nightjar activity on the wav files from both channels were classified by Song Scope using the recognizer. Before running the batch process we chose to save only the results that had a score above 40.0% and a quality above 0. The score value is on a scale from 0.00% to 100.0% and represents the statistical fit of the candidate nightjar vocalisation to the nightjar recognizer model. The quality value is on a scale from 0.00 to 99.99, and reflects how well a set of secondary parameters of the candidate nightjar vocalisation match with the training data used to build the recognizer. We first did a batch process with minimum score of 0% and minimum quality of 0 on three audio files known to have a substantial amount of nightjar vocalisations and ordered the results by score. We then looked through these results and noted the minimum score for audio files that included genuine nightjar vocalisations, which in our case was 40.0%. This score value was then used in the batch process which was run on all files. In consultation with Wildlife Acoustics Inc., we decided to keep the quality value at 0 to reduce false negatives. We listened to 20 hours of recordings (1% of the total) to estimate false negatives with these settings. The churring recognizer had a false negative rate of 4.4%, while the flight call recognizer had a false negative rate of 13.4%. In our 20-hour sample, false negatives were caused exclusively by either failing to recognize very distant nightjar vocalisations or, in the case of the flight calls, by simultaneous churring overlapping and obscuring the calls. Finally, all files in our sample where the recognizer did not detect a nightjar at all were verified as true negatives in our listening experiments. Thus, the recorders performed very well at detecting nightjar presence, with any false negatives restricted to the recognition of individual calls.

After the batch process finished, all positive results were manually verified, and false positives were deleted from the analysis (in total we found 766,275 false positives across all recorders). To understand the cause of these false positives, we manually investigated all cases where the recognizer reported more than 100 results in one 30-min file but where no churring or flight calls had been recognized (80.1% of all false positives). This included 84.3% of the false positives for the churring analysis, of which 99.2% were due to bad weather (heavy wind or rain), and 69.9% of the false positives for the flight call analysis, of which 88.7% due to bad weather (heavy wind or rain). False positives could be reduced by excluding recordings when there is bad weather. In addition, during a batch process, settings for score and/or quality could be increased to reduce the false positives (e.g. increasing the score value to 65.0% reduced our total false positives to 53,789) but this has a tradeoff with the false negatives as these will then be increased. The results from the churring recognizer were split into individual 60 second (s) sections. If any registration of a churring nightjar was made (of any duration) during a 60 s sample then that sample was recorded as a positive registration. For each 30 minutes of recording we listed the total number of positive 60 s samples. The results from the flight call recognizer were counted to give the total number of flight calls recognized in each 30 minutes segment of recording. The results were loaded in R 3.0.0 [16] for further analysis.

#### Comparison of traditional surveys with recorders

Data from traditional surveys were plotted in ArcGIS. We then determined the nearest recorder within a 500 m radius for each located nightjar registration and for each recorder, and each visit, we counted the number of nearest registrations. We transformed these data and the data from the recorders into presence/absence data. We compared the presence/absence data from the traditional surveys with the presence/absence data from the recorders with McNemar tests: that is, if one or more nightjars were located nearest to recorder A on visit A it was scored as a ‘1’; similarly if the audio recorders yielded data (either from the churring or the flight call analysis) for one or more nightjars, over a particular time period (see below), then it was scored as a ‘1’). The survey data were compared with the audio data that were obtained during weeks 1, 3, 5, and 7 (n = 22; we had four visits and six recorders. As recorder 6 was not deployed until week 4, the data from visit 1 and 2 near the subsequent location of this recorder could not be compared with any audio data).

Furthermore, we tested whether the survey data on the abundance of nightjars at each recorder was correlated with the amount of vocalisation that was recorded on the same recorder. A correlation would be expected if traditional human surveys were accurate and the amount of vocalisation could then be used to infer numbers of nightjars. For this, we fitted two generalised linear mixed models (GLMMs) with a Poisson error structure, with recorder as a random effect, and the amount of vocalisation (either churring or flight calls) as fixed effects. We compared the survey data with recordings made during the week after the survey (n = 22). For these tests the data were also split per recorder, per visit.

#### Based on the recorded data, when is it best to survey for nightjars?

To test what time during the night nightjars are most active, we split the data into three time periods: 22.00–00.00 (dusk), 00.00–02.30 (middle), 02.30–04.30 (dawn). We then compared the activity (churring or flight calls) recorded via a GLMM with a Poisson error structure. As the activity of nightjars differed between recorders, we fitted the different recorders as a random effect. Since the total recording time was different between recorders, we fitted this as an offset.

#### Comparison of recorder settings

We subsampled the data collected by the recorder to inform how to use the recorders. We were particularly interested in testing if the recorder could be set to record for shorter time periods while achieving similar results; this would save battery power so that the recorders could be left out for longer periods. Therefore, we compared the following settings that the recorder might be set to: (1) recording 10 mins every hour, (2) only dusk (22.00–00.00), (3) only the middle of the night (00.00–02.30) (4) only dawn (02.30–04.30), (5) dusk and dawn, and (6) recording all night. For this we selected the respective data from the data collected via the recognizers. For example (1) for every hour of recording the first 10 mins was selected (e.g. 22.00–22.10, 23.00–23.10) and the total number of churring mins and flight calls was calculated. We then calculated the total number of nights “nightjar_nights” that a nightjar was detected by each recorder (either via the churring or the flight call recognizer). We scored each night that a nightjar was detected as ‘1’ (nights with no nightjars were scored as ‘0’). We then fitted a generalised linear model with a Poisson error structure with the formula: nightjar_nights ∼ setting+offset(log(total_nights)), where the “total_nights” is the total number of nights that the recorder was on.

All GLMMs were fitted with the package lme4 [17]. Pairwise post-hoc analysis was done via the glht function in the multcomp package [18]. We used an alpha value of 0.05 to assess the significance of results. All statistical tests were performed in R version 3.0.0 [16].

## Results

### Comparison of traditional surveys with recorders

We compared the detection of nightjars via traditional human surveys with detection by bioacoustic recorders using the data recorded at the same time as the human surveys (22∶00–00∶00 on survey nights), and found that the recorders detected nightjars during five of the eleven survey periods, while the humans only detected nightjars during three of the eleven survey periods. When we looked at nightjar detection by the recorders during the whole survey night (22∶00–04∶30) we found that detection by the recorders increased to eight of the eleven survey periods. Moreover, when we compared the results of the human surveys with recordings made during the whole week of the survey, we found that the recorders detected nightjars during 19 of 22 survey periods, while surveyors detected nightjars on only six of these occasions; human surveyors never detected a nightjar that the recorders failed to detect. This equates to a 217% increase in detection of nightjars using bioacoustic devices when compared with human surveyors.

**Figure 1:**
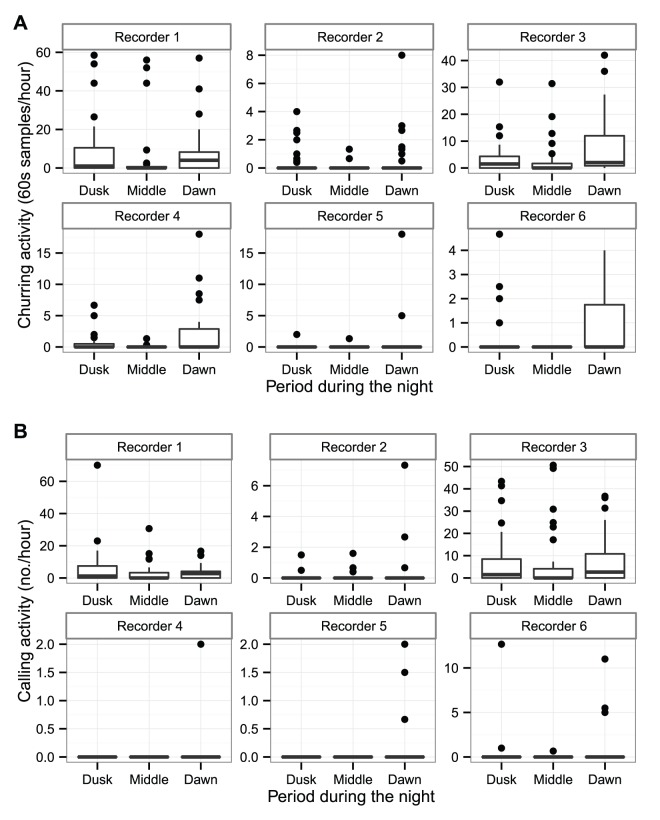
Nightjar activity at three different periods during the night as detected by automated recorders. These boxplots display (a) churring activity (measured in minutes of activity per hour) and (b) flight calling activity at six different automated recorders during the period of the survey: Dusk, 22.00–00.00; Middle, 00.00–02.30; Dawn, 02.30–04.30. The y-axes are distinct for each recorder because the amount of activity varied greatly according to location (and local abundance of nightjars). Most of the churring activity was recorded during the dawn period and the least amount of activity was during the middle of the night. There was no difference in flight calling activity between the three periods.

We found no correlation between the abundance of nightjars found by the traditional surveys at each recorder and the amount of vocalisation (either churring activity or flight calling activity) that was recorded on the same recorder (churring activity: estimate = 0.000, std error = 0.004, z-value = 0.087, p-value = 0.931; flight calling activity: estimate = −0.025, std error = 0.023, z-value = −1.093, p value = 0.274). For example, at recorder 1 there was a substantial amount of nightjar activity during dusk and dawn (see Figure 1), but nightjars were not detected during any of the four traditional human surveys. We therefore judge it unwise to provide accurate estimates of nightjar numbers in our study area. However, we can give a crude estimate based on the human surveys via methodology described by Gilbert et al [15]. This yielded a count of eight different churring males, but it is very likely to be an underestimate.

### Based on the recorded data, when is it best to survey for nightjars?

We found that nightjars are most active at dawn and least active during the middle of the night based on their churring activity (see Figure 1a). Churring activity during dawn was significantly greater than during dusk or middle of the night (dusk-dawn: estimate = −0.531, std error = 0.048, z-value = −11.172, p<0.001; middle of night-dawn: estimate = −1.018, std error = 0.052, z-value = −19.729, p<0.001). There was more churring activity during dusk than during the middle of the night (estimate = −0.487, std error = 0.054, z-value = −9.075, p<0.001). We found no significant difference in flight calling activity during the three periods (dusk-dawn: estimate = −0.009, std error = 0.053, z-value = −0.169, p = 0.984; middle of night-dawn: estimate = 0.003, std error = 0.051, z-value = 0.053, p = 0.998; middle of night-dusk: estimate = 0.012, std error = 0.046, z-value = 0.253, p = 0.965; see Figure 1b).

### Comparison of recorder settings

**Figure 2:**
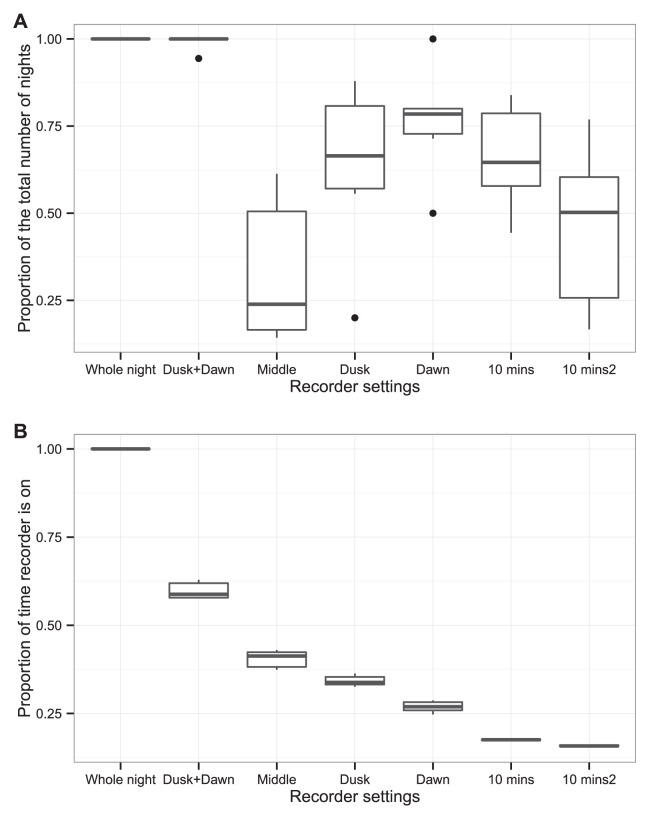
Proportion of the total number of nights nightjars were detected using different recorder settings. (a) We plotted the following recorder settings: dusk (22.00–00.00), middle of the night (00.00–02.30), dawn (02.30–04.30), dusk and dawn combined, and two random samples of ten minutes per hour (“10 mins” and “10 mins2”), and compared these to the total nights nightjars were detected when the recorders were left on during the whole night. (b) The relative length of each of these recording periods compared to recording the whole night. Our results indicate that most nightjar activity occurs during dusk and dawn; as a result, activating the recorders during these periods captures almost all (98.8%) of the nightjar activity, even though these periods make up only 60% of the night. The variability among the two 10-minute subsamples suggests that this is an unreliable sampling strategy for detecting nightjar activity.

We found that detection of nightjar activity varied among the different recorder settings. Having the recorder on only at dawn identified many of the nights that nightjars were active (75% in comparison to recording the whole night), while the recording time was substantially decreased (27% in comparison to recording the whole night). In addition, recording at both dawn and dusk also performed well. This setting detected almost all (98.8%) of the nights that there was some nightjar activity while only recording for 60% of the night. These results can be explained by our finding that nightjars are most active at dawn and at dusk. Setting the recorder to 10 mins every hour throughout the night had a varied performance; we tested two random sets of 10 mins every hour (e.g. 22.00–22.10, 23.00–23.10, etc and 22.30–22.40, 23.30–23.40, etc) and one performed significantly worse than the other (p = 0.036). Recording only in the middle of the night (thus excluding dawn and dusk) had the worst performance of all the settings (see Table 1 and Figure 2).

**Table 1:** Comparison of nightjar detection by different recorder settings.

Comparison	Estimate	Std Error	Z value	p-value adjusted
Dusk+Dawn – whole night	−0.016	0.081	−0.201	0.999
**Middle – whole night**	**−0.868**	**0.105**	**−8.268**	**<0.001**
**Dusk – whole night**	**−0.324**	**0.088**	**−3.698**	**0.004**
Dawn – whole night	**−**0.251	0.087	**−**2.877	0.060
**10 mins – whole night**	**−0.374**	**0.089**	**−4.209**	**<0.001**
**10 mins2– whole night**	**−0.696**	**0.098**	**−7.083**	**<0.001**
**Middle – Dusk+Dawn**	**−0.852**	**0.105**	**−8.095**	**<0.001**
**Dusk – Dusk+Dawn**	**−0.307**	**0.088**	**−3.501**	**0.008**
Dawn – Dusk+Dawn	**−**0.235	0.087	**−**2.682	0.101
**10 mins – Dusk+Dawn**	**−0.358**	**0.089**	**−4.013**	**0.001**
**10 mins2– Dusk+Dawn**	**−0.680**	**0.099**	**−6.899**	**<0.001**
**Dusk – Middle**	**0.545**	**0.111**	**4.919**	**<0.001**
**Dawn – Middle**	**0.617**	**0.110**	**5.591**	**<0.001**
**10 mins – Middle**	**0.494**	**0.112**	**4.425**	**<0.001**
10 mins2 **−** Middle	0.172	0.119	1.439	0.777
Dawn – Dusk	0.073	0.094	0.775	0.987
10 mins – Dusk	**−**0.050	0.095	**−**0.525	0.998
**10 mins2– Dusk**	**−0.373**	**0.104**	**−3.570**	**0.007**
10 mins – Dawn	**−**0.123	0.095	**−**1.292	0.853
**10 mins2– Dawn**	**−0.446**	**0.104**	**−4.280**	**<0.001**
**10 mins2–10 mins**	**−0.323**	**0.105**	**−3.058**	**0.036**

The mean detection of nightjars by the different recorder settings is compared via Tukey Contrasts for the fitted Generalised Linear Model. The detection of nightjars is compared for the different recorder settings. Adjusted p-values are reported (single-step method) and significant p-values are given in bold. The different settings were: recording 10 mins every hour (“10 mins” & “10 mins2”), only dusk (22.00–00.00), only the middle of the night (00.00–02.30), only dawn (02.30–04.30), dusk and dawn (“Dusk+Dawn”), and recording all night (“whole night”).

## Discussion

We compared the detection of nightjars from bioacoustic recorders deployed at the same time as experienced field surveyors, and found that recorders detected more nightjars (five out of eleven survey periods versus three out of eleven survey periods respectively). However, anyone wishing to detect nightjar presence with a recorder is unlikely to deploy the recorder for a few hours; instead, typical usage would involve leaving the device in situ for an extended period. When we compared the results from recorders deployed for a week, we found that bioacoustic recorders were significantly (and substantially) better at detecting nightjars than human surveyors. The recorders detected nightjars during 19 of 22 survey periods, while surveyors detected nightjars on only six of these occasions. In addition, there was no correlation between the amount of vocalisation detected by the recorders and the abundance of nightjars recorded by human surveyors – a correlation which would be expected if human surveyors detected most of the birds present. The recordings revealed that nightjars were most active just before dawn and least active during the middle of the night. Recording at both dusk and dawn, or only at dawn, would give reasonably high levels of detection of nightjar activity while only recording for 60% and 27% of the night respectively.

While nightjars are not listed as one of the species that are difficult to survey in guidance by JNCC [5], our analyses reveal a major difference in detection by humans and recorders. This indicates that current survey methods for nightjars could be substantially improved, especially since we performed four surveys rather than the minimum of two surveys recommended by Gilbert et al. [15]. As nightjars are listed in Annex I of the Bird Directive, quantifying detectability for this species is important [6], with several Special Protection Areas (SPA) in the UK designated specifically for their breeding nightjar populations. SPAs in the UK are designated if an area is regularly used by >1% of the national population of a species that is listed on Annex I of the Bird Directive in any season. Our analyses indicate that bioacoustic recorders provide more accurate information on nightjar distributions, and could therefore be used to help better define SPAs.

One of the possible reasons for low detection by human surveyors could be that nightjars vocalise for short periods (e.g. we found vocalisations of <10 mins every hour (see Figure 1a)). Nightjars are a nocturnal species and thus surveys will be performed in low-light conditions, with surveyors relying mostly on their hearing to detect nightjars. The length of time that a human surveyor passes the location of a nightjar is probably of short duration minimising the probability of detecting a vocalising nightjar. As nightjars vocalise for short periods, increasing the frequency and/or length of stopping and listening for nightjars during a human survey may not significantly improve detection by human surveyors.

We found that nightjars are most active just before dawn or just after dusk. Therefore, our results confirm and extend the previous findings of Cadbury [19], which were based on only a limited number of surveys. Our analysis showed that recorders could be set to record for shorter periods of time while achieving similar results, if knowledge of peak activity times is available. This would save on battery life and data storage. As a result, the recorders could be left out for longer before the batteries would run out of power or before the data storage is full. For example, recording for two hours at dawn using SM2+ units without GPS would result in almost 13 nights of deployment (average battery life of 25.4 hours divided by two hours). Note that our average battery life of 25.4 hours results from our use of rechargeable batteries; if desired, recording time could be increased through the use of alkaline batteries or an external power supply such as a solar panel or a 6V or 12V battery source. We were not able to determine when nightjars are most active during the breeding season (e.g. during territory establishment or chick rearing). To answer this question, data from multiple years are needed as weather and other factors might have affected nightjar activity.

### Advantages of using bioacoustic recorders

Our results suggest that bioacoustic recorders could be a very effective survey method for a variety of reasons. Firstly, surveys for species that do not vocalise regularly have a low accuracy as we have shown here in the case of nightjars. Secondly, surveying with bioacoustic recorders causes less disturbance than traditional surveys, as there is only the initial visit of deploying and picking up the recorders which could be done at a time when the species of interest is not active. Therefore, bioacoustic recorders could be used for species that are affected by human disturbance such as the capercaillie, short-eared owl and peregrine falcon [5]. Thirdly, they can be of benefit when surveys are to be carried out in remote or difficult to access areas, as visits need only be made when deploying and picking up the recorders or replacing the batteries. Fourthly, there is no need for specialist surveyors to deploy or retrieve the recorders. Local people who know the area could deploy the recorders at a given location without the need for any detailed knowledge of the species of interest. Data could be analysed either by a specialist or, for species where a recognizer has been built (which will become more common as the use of automated recognition increases), by anyone with access to a personal computer. Currently, surveys for species that require a specialist include aquatic warbler, capercaillie, and goshawk [5]. However, it may be more difficult to use automated classification for the vocalisations of these species as these vocalisations might be more complex than those of the nightjar. In addition, there might be more background noise interference since these species are active during the daytime, but noise filters could be applied to the recordings to reduce this interference. A fifth advantage is that the cost of surveying using bioacoustic recorders is cheaper. We estimated the costs for an ecological consultancy company to carry out surveying work on one of the two sites from this study using field surveyors and automated bioacoustic recorders. A field survey approach would require four visits and would require 16 hours (based on two surveyors for Health and Safety reasons and assuming two hours per visit per person). In contrast, the approach using automated recorders would require three visits during the daytime: one visit to deploy the devices, one to replace the batteries and another visit to collect the devices. We estimate that this would require three hours (assuming one fieldworker). This needs to be offset by costs for processing the data from the recorders which we estimate to be two hours per device. On our sites we used three devices per site so assuming this, the processing time would total six hours. Thus, based on our theoretical example, a saving of seven hours work would be made using automated bioacoustic recorders, which saves 44% of time. Based on UK industry standards for an Ecologist or Senior Ecologist, this would be a saving of approximately £350 (based on £50/hour; source: Chartered Institute of Ecology and Environmental Management, http://www.cieem.net). Of course, the automated recorders would also provide a much richer source of survey data as they would be running for much longer periods than the field surveyors are present.

### Further work on nightjars

This work has demonstrated the power of using bioacoustic recorders for determining the presence/absence of nightjar within a survey area. Traditionally, the presence of churring has been used as an indication of breeding activity and has been extrapolated to infer the numbers of breeding pairs present within an area. However, churring *per se* is not an indication of pairing or breeding. It has been postulated (Andrew Lowe pers. comm.) that the call structure of the male nightjar is modified once a territory is established and mating is successful. If this hypothesis can be confirmed and the pre- and post-mating call can be quantified using bioacoustics, it may be possible to gain a more accurate measure of the breeding populations of this species, which to our knowledge has not been tried for this or any other species.

### Conclusions

Here we have shown that bioacoustic recorders offer substantial improvements (217% increase in detection) over human surveyors in the detection of nightjars (an infrequently vocalising nocturnally active species) when deployed throughout the night. While previous studies have used bioacoustic recorders, they used interpreters to listen to the recorded audio files and classify the species on the recordings instead of automated classification. Listening to the audio files is very labour intensive, and automated classification could save a lot of time, especially when leaving the recorders out for longer periods [9]. These previous studies detected similar numbers and species as observers conducting point counts in the field [8], [11], [20] but only recorded for the duration of a traditional point count survey and did not investigate if recording for longer periods of time would increase performance, as is now possible with deployable recorders. Here we have shown that the accuracy of the recorders increases when they are deployed for a longer period of time. For example, when looking at the data recorded at the same time as the human surveys (22.00–00.00 on survey nights) nightjars were detected on five out of eleven instances (45%) by the recorders. However, the detection increased to eight out of eleven instances (73%) when we increased the selected recording time to the whole night of the survey (22.00–04.30 on survey nights; note that a 100% detection rate is likely unachievable as nightjars did not make regular use of some of the areas we surveyed). In addition, as we have performed line transect surveys, we also show for the first time that bioacoustic recorders can be used instead of line transect surveys and not only to replace point counts.

Our study has implications for a range of other species, especially for species that are difficult to detect by traditional survey methods. For these species, we show that there is great potential to increase detection by using automated bioacoustic recorders. This is especially important when information on species presence and abundance is used to inform conservation.

## Supporting Information

Figure S1 **Map of study sites displaying the locations of the bioacoustic recorders and the survey route followed by human surveyors.** Two Forestry Commission sites in Northumberland, UK, were surveyed: (a) Slaley Forest and (b) Fourlaws. Survey methods followed the methods described in Gilbert et al. [15]. At each site three full spectrum recorders (SM2+, Wildlife Acoustics Inc.) were placed, one per km^2^ (six in total), during the same period as the traditional site surveys (between mid-June and the end of July 2012).
(PDF)

Table S1 **Number of recordings for each 30-minute interval throughout the survey period.**
(DOC)

Table S2 **Recorder sampling scheme over the survey period.**
(DOC)

Table S3 **Churring recognizer configuration settings.**
(DOC)
