# Supplementary figures and images for: The Use of Automated Bioacoustic Recorders to Replace Human Wildlife Surveys: An Example Using Nightjars
Source: PLoS One. 2014 Jul 16;9(7):e102770. doi: 10.1371/journal.pone.0102770 (PMC4100896; doi:10.1371/journal.pone.0102770)

## A. Slaley Forest

- Location of recorder

— Survey route

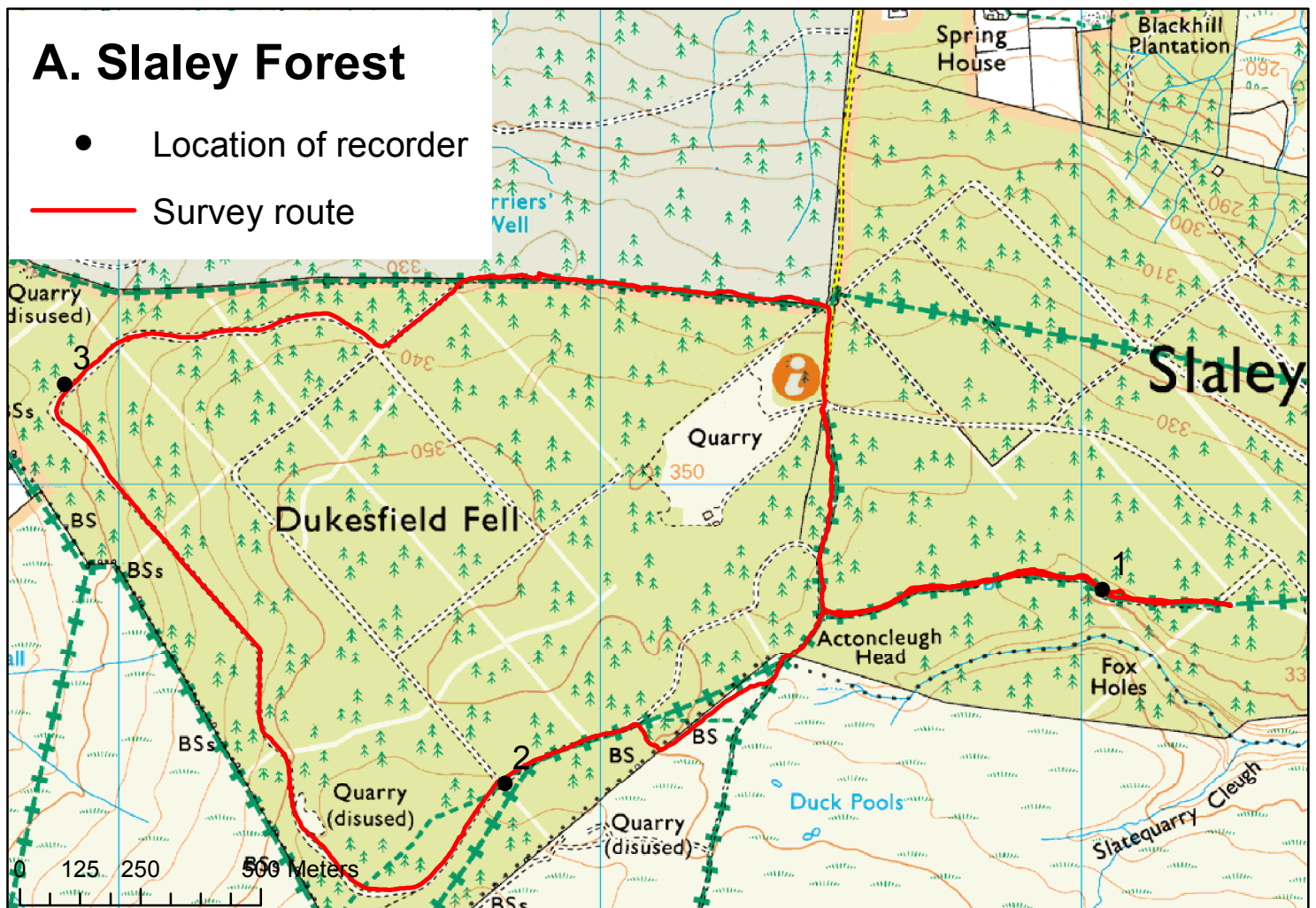

## B. Fowlaws

- Location of recorder

— Survey route

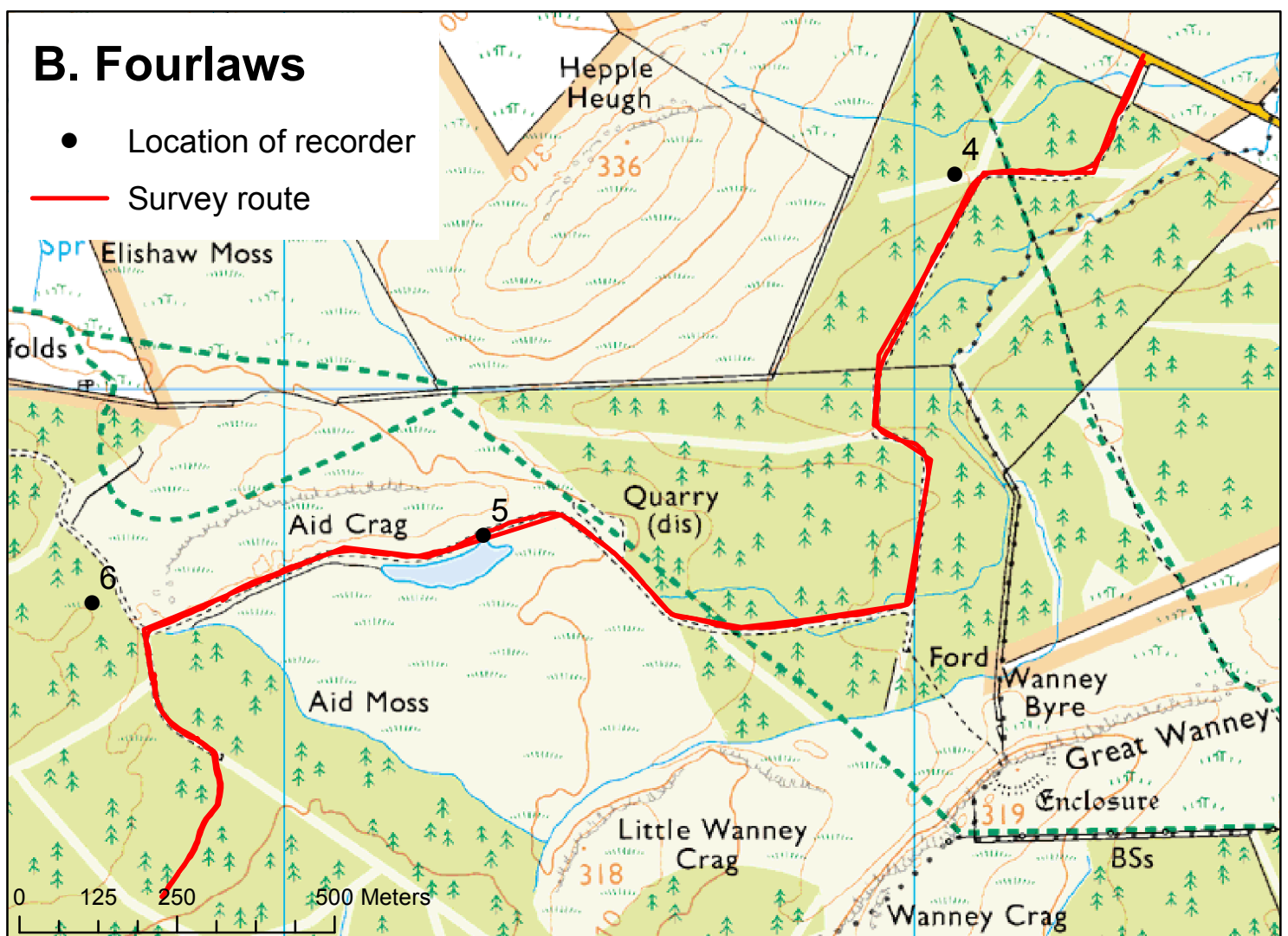

Supplement: Figure S1 — Map of study sites displaying the locations of the bioacoustic recorders and the survey route followed by human surveyors. Two Forestry Commission sites in Northumberland, UK, were surveyed: (a) Slaley Forest and (b) Fourlaws. Survey methods followed the methods described in Gilbert et al. [15]. At each site three full spectrum recorders (SM2+, Wildlife Acoustics Inc.) were placed, one per km2 (six in total), during the same period as the traditional site surveys (between mid-June and the end of July 2012). (PDF) [file pone.0102770.s001.pdf]
