# Supplementary material for: The Use of Automated Bioacoustic Recorders to Replace Human Wildlife Surveys: An Example Using Nightjars
Source: PLoS One. 2014 Jul 16;9(7):e102770. doi: 10.1371/journal.pone.0102770 (PMC4100896; doi:10.1371/journal.pone.0102770)
Supplement: Table S1 — Number of recordings for each 30-minute interval throughout the survey period. (DOC) [file pone.0102770.s002.doc]

**Table S1.** **Number of recordings for each 30-minute interval throughout the survey period.**

| Time | Recorder 1 | Recorder 2 | Recorder 3 | Recorder 4 | Recorder 5 | Recorder 6 | Total |
| --- | --- | --- | --- | --- | --- | --- | --- |
| 22:00-22:30 | 29 | 30 | 29 | 17 | 18 | 17 | 140 |
| 22:30-23:00 | 37 | 34 | 34 | 27 | 27 | 17 | 176 |
| 23:00-23:30 | 37 | 34 | 34 | 26 | 26 | 17 | 174 |
| 23:30-00:00 | 35 | 34 | 34 | 26 | 26 | 17 | 172 |
| 00:00-00:30 | 34 | 34 | 34 | 26 | 26 | 17 | 171 |
| 00:30-01:00 | 34 | 34 | 34 | 25 | 26 | 16 | 169 |
| 01:00-01:30 | 32 | 33 | 33 | 25 | 26 | 16 | 165 |
| 01:30-02:00 | 32 | 33 | 32 | 25 | 25 | 16 | 163 |
| 02:00-02:30 | 32 | 33 | 31 | 24 | 24 | 15 | 159 |
| 02:30-03:00 | 30 | 32 | 30 | 24 | 24 | 15 | 155 |
| 03:00-03:30 | 24 | 28 | 28 | 20 | 20 | 15 | 135 |
| 03:30-04:00 | 18 | 20 | 18 | 10 | 12 | 9 | 87 |
| 04:00-04:30 | 18 | 17 | 18 | 9 | 11 | 9 | 82 |

We deployed 6 SM2+ (Wildlife Acoustics Inc.) automated bioacoustic recorders (one per km^2^) at two sites in Northumberland between mid-June and the end of July 2012. Recorders 1-3 were placed at Slaley forest and recorders 4-6 at Fourlaws. The recorders were set to record throughout the night between 22.00 and 04.30 the following morning on both channels with a gain of +48dB and sampling rate of 44100Hz. This table shows the number of 30-minute recordings that were obtained for each recorder and time period; this number varies due to variation in battery use, which depends on local sound levels.
