# Supplementary material for: The Use of Automated Bioacoustic Recorders to Replace Human Wildlife Surveys: An Example Using Nightjars
Source: PLoS One. 2014 Jul 16;9(7):e102770. doi: 10.1371/journal.pone.0102770 (PMC4100896; doi:10.1371/journal.pone.0102770)
Supplement: Table S2 — Recorder sampling scheme over the survey period. (DOC) [file pone.0102770.s003.doc]

**Table S2. Recorder sampling scheme over the survey period.**

| **Sunrise** | **Sunset** | **Week** | **Recorder 1** | **Recorder 2** | **Recorder 3** | **Recorder 4** | **Recorder 5** | **Recorder 6** |
| --- | --- | --- | --- | --- | --- | --- | --- | --- |
| 4:28 | 9:45 | 1 | 22:00-02:30* | 22:00-03:00* | 22:00-03:00* | 22:30-03:00* | 22:30-03:00* |  |
| 4:27 | 9:49 | 2 | 22:30-03:00 | 22:30-03:30 | 22:30-03:30 | 22:30-03:30 | 22:30-03:30 |  |
| 4:32 | 9:48 | 3 | 22:30-03:00* | 22:30-03:30 | 22:30-03:30 | 22:30-03:30* | 22:30-03:30* |  |
| 4:38 | 9:44 | 4 | 22:00-03:30 | 22:00-03:30 | 22:00-03:30 | 22:00-03:30 | 22:00-03:30 | 22:00-03:30 |
| 4:48 | 9:36 | 5 | 22:00-04:30 | 22:00-04:30 | 22:00-04:30 | 22:00-04:30 | 22:00-04:30 | 22:00-04:30 |
| 4:57 | 9:27 | 6 | 22:00-04:30 | 22:00-04:30 | 22:00-04:30 | 22:00-04:30 | 22:00-04:30 | 22:00-04:30 |
| 5:09 | 9:16 | 7 | 22:00-04:30 | 22:00-04:30 | 22:00-04:30* | 22:00-04:30* | 22:00-04:30* | 22:00-04:30 |
| 5:18 | 9:06 | 8 | 22:00-04:30 | 22:00-04:30 | 22:00-04:30 |  |  |  |
| 5:27 | 8:56 | 9 | 22:00-04:30 | 22:00-04:30 | 22:00-04:30 |  |  |  |

We deployed 6 SM2+ (Wildlife Acoustics Inc.) automated bioacoustic recorders (one per km^2^) at two sites in Northumberland between mid-June and the end of July 2012. Recorders 1-3 were placed at Slaley forest and recorders 4-6 at Fourlaws. The recorders were set to record throughout the night between 22.00 and 04.30 the following morning on both channels with a gain of +48dB and sampling rate of 44100Hz. Each week the recorders were active until the batteries ran dry, which on average was four nights (range 3-5 nights). The traditional surveys took place at the beginning of week 1, 3, 5 and 7. An asterik (*) denotes whether the recorders were on during the night of the traditional survey.
