# Supplementary material for: The Use of Automated Bioacoustic Recorders to Replace Human Wildlife Surveys: An Example Using Nightjars
Source: PLoS One. 2014 Jul 16;9(7):e102770. doi: 10.1371/journal.pone.0102770 (PMC4100896; doi:10.1371/journal.pone.0102770)
Supplement: Table S3 — Churring recognizer configuration settings. (DOC) [file pone.0102770.s004.doc]

**Table S3. Churring recognizer configuration settings.**

| **Setting** | **Configuration 1** | **Configuration 2** | **Configuration 3** | **Final configuration** |
| --- | --- | --- | --- | --- |
| Maximum complexity | 32 | 42 | 16 | 16 |
| Maximum resolution | 6 | 8 | 11 | 15 |
| Sample rate | 16 kHz | 6 kHz | 8 kHz | 8 kHz |
| FFT size | 256 | 32 | 64 | 64 |
| FFT overlap | ½ | ½ | ½ | ½ |
| Frequency minimum | 9 | 6 | 8 | 8 |
| Frequency maximum | 49 | 10 | 20 | 18 |
| Background filter | 2s | 1s | 2s | 1s |
| Maximum syllable length | 48 ms | 27 ms | 100 ms | 148 ms |
| Maximum syllable gap | 48 ms | 32 ms | 100 ms | 148 ms |
| Maximum song length | 24 ms | 60000 ms | 10392 ms | 5980 ms |
| Dynamix range | 20 | 10 | 10 | 10 |
| Algorithm | 2.0 | 2.0 | 2.0 | 2.0 |

We chose the initial recognizer configuration (Configuration 1) by manually experimenting with recognizer parameters in Song Scope until the true positives in our training dataset were correctly recognized. In Song Scope, when the log frequency scale with normalized power levels is selected, the program displays the recognized sections of the training data with hot colours. When recognizer settings are altered, Song Scope updates these recognized sections.

Configuration 2: FFT size and sample rate was reduced in order to get a better temporal resolution of the pulses that make up the churring of the nightjar. When FFT size and sample rate are adjusted the frequency minimum and maximum need to be adjusted as well in order to select the same frequency bandwidth. In addition, maximum song length was increased as the length of nightjar churring is long. Furthermore, we reduced the dynamic range in order to reduce the background interference.

Configuration 3: we reduced the maximum song length as alternative configuration 2 was giving too many false negatives. We also reduced the maximum complexity. Furthermore, we increased the maximum syllable length and gap. Alternative configuration 3 gave much better results (fewer false negatives and positives) than the previous two.

Final configuration settings: through further experimentation, we found that adjusting the maximum syllable length, maximum syllable gap and maximum song length provided additional improvements.
